# Comparison of Pregabalin and Sodium Valproate in Migraine Prophylaxis: A Randomized Double-Blinded Study

**Published:** 2018

**Authors:** Omid Hesami, Mohammad Reza Shams, Ladan Ayazkhoo, Farhad Assarzadegan, Behnam Safarpour Lima, Hosein Delavar Kasmaei, Mohammad Sistanizad

**Affiliations:** a *Department of Neurology, Imam Hossein Medical and Educational Center, Shahid Beheshti University of Medical Sciences, Tehran, Iran. *; b *Department of Neurology, Besat Hospital, Military University of Medical Sciences, Tehran, Iran. *; c *Department of Clinical Pharmacy, Faculty of Pharmacy, Shahid Beheshti University of Medical Sciences, Tehran, Iran. *; d *Department of Neurology, Shohada Medical and Educational Center, Shahid Beheshti University of Medical Sciences, Tehran, Iran. *; e *Department of Pharmaceutical Care Unit, Imam Hossein Medical and Educational Center, Shahid Beheshti University of Medical Sciences, Tehran, Iran.*

**Keywords:** Migraine, Prophylaxis, Pregabalin, Valproate sodium, Headache

## Abstract

Patients suffering from headache, particularly migraine type, are among the most dissatisfied patients. The aim of this study was comparing the efficacy of pregabalin with valproate sodium, in preventing migraine headache.

In a randomized, double-blinded study, adult patients eligible for prophylactic treatment (*i.e.*, patients with 4-15 attacks per month in last two months) were recruited. Patients’ demographic data, duration of symptoms, headache frequency (attacks per month) and intensity (based on visual analogue scale) and also drugs used to relief headache were recorded. The patients were randomly assigned to two groups; valproate sodium (200 mg two times daily) and pregabalin (50 mg two times daily). The patients were examined by neurology specialist monthly for three months and the related data were recorded. The Data were analyzed using SPSS version 21, with related statistical tests.

Total number of 140 patients with recurrent migraine were entered into the study. Sixty-nine patients were assigned to group A and 71 to group B by the randomizing table. Inter-group analysis of data in two arms of the study showed that two medications were equally effective except that pregabalin was not significantly effective in reducing number of attacks during first month of therapy compared to baseline. This differences were not significant at second and third month of the study.

Our study showed that pregabalin, has comparable efficacy with valproate sodium in reducing migraine frequency, intensity, and duration of attacks and could be an alternative for migraine prophylaxis.

## Introduction

Headache, one of the most common patient complaints in neurologists’ offices and the most common pain complaint seen in family practice, accounts for 10 million office visits a year (1, 2). Most headaches are of the primary type (*e.g.*, migraine and tension-type headache). An estimated 6% of men and 15% to 17% of women in the United States have migraine, but only 3% to 5% of them receive preventive therapy (3). About half of migraine patients stop seeking care for their headaches, partly because they are dissatisfied with therapy. Indeed, public surveys indicate that headache sufferers are among the most dissatisfied patients. In addition to being dissatisfied with their care, many migraineurs report significant disability, impaired quality of life, and impaired work-related productivity (4).

Following appropriate management of acute migraine, patients should be evaluated for initiation of preventive therapy. The goal of the preventive therapy is to improve patients’ quality of life by reducing migraine frequency, severity, and duration, and by increasing the responsiveness of acute migraines to treatment. Therapy should be initiated with medications that have the highest levels of effectiveness and the lowest potential for adverse reactions (5, 6). Numerous medications have been used to prevent migraine headaches, including b-blockers, calcium-channel blockers, anticonvulsants, SSRIs, TCAs, and nonsteroidal anti-inflammatory drugs (4-7) but, side effects, poor compliance, and disappointing outcomes are common. Cognitive symptoms also emerge frequently in this context, either as a consequence of chronic pain (2, 8 and 9) or of the medications used to treat refractory migraine (10, 11).

Migraine and epilepsy share several clinical features, and epilepsy is a comorbid condition of migraine (6, 12 and 13). With this concept, anticonvulsants are among most studied and recommended drugs in preventive treatment of different types of headaches (12, 14 and 15) and sodium valproate and topiramate have been approved by the US Food and Drug Administration (FDA) for migraine prevention (6, 16).

Pregabalin, is an anticonvulsant drug which binds to alpha2-delta subunit of voltage-gated calcium channels within the CNS and modulates calcium influx at the nerve terminals, and inhibits excitatory neurotransmitter release including glutamate, norepinephrine, serotonin, dopamine, substance P, and calcitonin gene-related peptide (17, 18). These mechanisms are coherent with the existing data regarding glutamatergic mechanisms in migraine physiopathology. High plasma and cerebrospinal fluid levels of glutamate level have been shown in patients with migraine (19, 20), and one study has revealed a significant decrease of plasma glutamate concentrations after prophylactic migraine treatment (21). There is no available trial of pregabalin use in the migraine prophylaxis in adults (22).

The primary aim of this study was comparing the efficacy of pregabalin with valproate, as an FDA approved prophylactic agent, in preventing migraine headache. Secondarily, we aimed to investigate the safety and the tolerability of pregabalin.

## Experimental

This study was a randomized, double-blind clinical trial to compare the effect of pregabalin and sodium valproate in preventing migraine headache. The study was performed between September 2014 and November 2015 in a teaching hospital affiliated to Shahid Beheshti University of medical sciences, Tehran, Iran. Diagnosis of migraine was done based on International Headache Criteria (ICHD III) (23) by a neurology specialist. Adult patients (age between 18 to 50 year) eligible for prophylactic treatment (*i.e.*, patients with 4-15 attacks per month in last two months) were recruited. Exclusion criteria included other types of headache; administration of prophylactic treatment for migraine; pregnancy; breast feeding; liver disease (child-pough score B or C) and sensitivity to pregabalin or Sodium valproate. Informed consent was obtained from the patient before entrance to the study.

**Table 1:** Demographic data of the patients.

		**Valproate (n = 46)**	**Pregabalin (n = 42)**	***P*** **-value**
Mean age, Y ± SD		35.48 ± 12.25	37.5 ± 9.86	NS
Gender (F)		43	36	NS
Weight	Male	65 ± 8.6	68.83 ± 8.15	NS
	Female	65.88 ± 10.25	67.53 ± 13.74	NS
Married		36	31	NS
Education level	uneducated	1	0	NS
	Below diploma	37	34	
	University educated	7	7	
Headache	Duration (Mean hour ± SD)	20.24 ± 19.1	20.83 ± 20.02	0.892
	No. of attacks/month (Mean ± SD)	10.19 ± 3.05	10.42 ± 3.07	0.727
	Intensity	7.51 ± 1.91	7.28 ± 1.57	0.556

**Table 2:** Intragroup analysis of duration, numbers and intensity of headaches at baseline and in monthly basis between two groups

Parameter		Valproate sodium (n = 46)		Pregabalin (n = 42)		*P*
		Mean ± SD	Median (Range)	Mean ± SD	Median (Range)	
**Duration of attack**	Baseline	20.24 ± 19.1	12(3 to 72)	20.83 ± 20.02	12(3 to 72)	0.892
	Month1	6.12 ± 5.24	5(0 to 24)	8.49 ± 7.67	7(0 to 31)	0.11
	Month2	3.92 ± 3.1	4(0 to 13)	5.5 ± 6.56	3.5(0 to 30)	0.195
	Month3	3.87 ± 4.34	3(0 to 20)	4.86 ± 6.46	3.5(0 to 30)	0.436
**Number of Attack**	Baseline	10.19 ± 3.05	10(5 to 14)	10.42 ± 3.07	10(4.5 to 14)	0.727
	Month1	6.12 ± 5.24	5(0 to 24)	8.49 ± 7.67	7(0 to 31)	0.11
	Month2	3.92 ± 3.1	4(0 to 13)	5.5 ± 6.56	3.5(0 to 30)	0.195
	Month3	3.87 ± 4.34	3(0 to 20)	4.86 ± 6.46	3.5(0 to 30)	0.436
**Intensity of attack**	Baseline	7.51 ± 1.91	8(4 to 10)	7.28 ± 1.57	8(4 to 10)	0.556
	Month1	4.88 ± 1.98	4.57(0 to 9)	4.75 ± 2.15	4.57(0 to 10)	0.788
	Month2	4.5 ± 2	4.4(0 to 8)	3.99 ± 2.17	4(0 to 8)	0.296
	Month3	3.8 ± 2.17	4(0 to 7.2)	3.69 ± 2.64	4(0 to 10)	0.847

**Figure 1:**
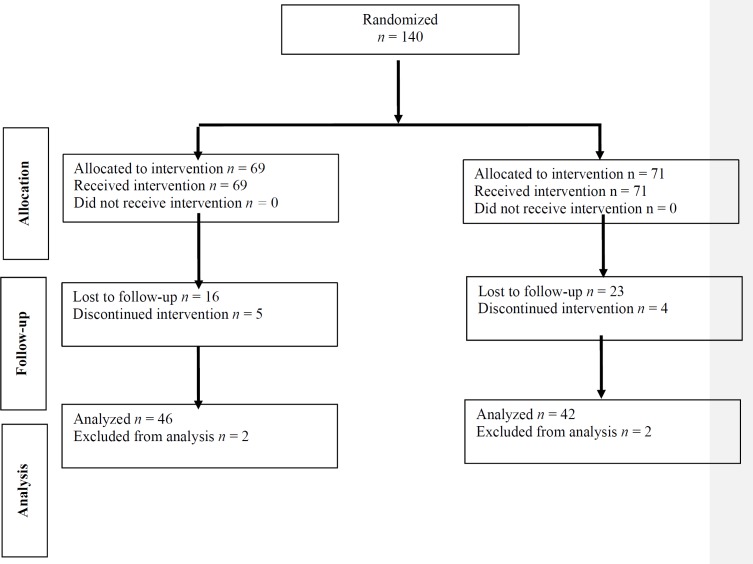
Consort chart of the study.

**Figure 2:**
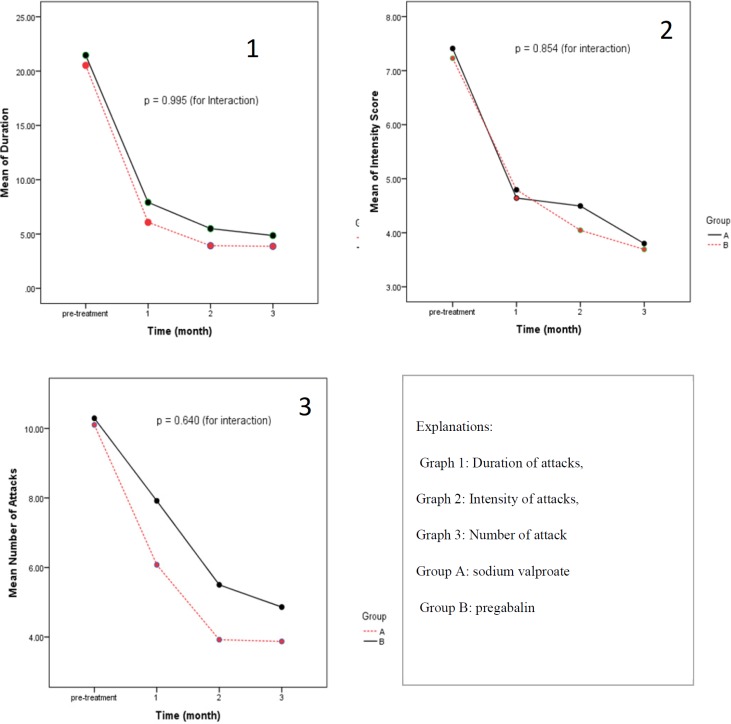
Duration, intensity and number of attacks in two arms of the study.

After signing the consent, patients’ demographic data containing sex, age, occupation, education level, neurologic exam data (including CT scan or MRI if existed), symptom duration, headache frequency (attacks per month) and intensity (based on visual analogue scale) as well as drugs used to relief headache were recorded.

Patients were randomly assigned to two groups; valproate sodium (200 mg two times daily) and pregabalin (50 mg two times daily) groups with completely identical tablets and containers without label, marked by codes (A or B) that was anonymous to research team till the end of the study. The patients were then followed for 3 months on monthly basis. 

The headache frequency and intensity, dose of analgesics were recorded. During monthly follow-up visits, a physical and neurologic exam was performed again and the patients were also asked about possible side effects. All analysis was performed using SPSS version 21, with related statistical tests. For all analysis,* P-*values less than 0.05 were considered statistically significant.

The study protocol was registered, reviewed, and approved by the Iranian Registry of Clinical Trials (IRCT), with registry number of IRCT2012070310178N1. IRCT is listed as a primary registry at the WHO International Clinical Trials Registry Platform

## Results

Total number of 140 patients with recurrent migraine were entered into the study. Sixty-nine patients were assigned to group A and 71 to group B by the randomizing table. Figure 1 shows the consort chart of the study.

*Demographic Data*

Eighty-eight patients (79 females and 9 men) with average age of 36 ± 11 were completed the study, 46 patients in group A (sodium valproate) and 42 in group B (pregabalin). Group A consisted of 43 women and 3 men with average age of 35.48 ± 12.25 and group B, 36 women and 6 men with average age of 37.5 ± 9.86 without statistically significant differences. None of the patient had an abnormal neurologic examination on each 4 visits (baseline and monthly for 3 months). Table 1 shows patients’ demographic data.

The effects of pregabalin and valproate sodium on duration, number and intensity of attacks were evaluated after 1, 2, and 3 months of receiving the medications. There were no statistically significant differences in measured parameters between two arms of the study. The results are shown in Table 2.

Inter-group analysis of data in two arms of the study showed that two medications were equally effective except that pregabalin was not significantly effective in reducing number of attacks during first month of therapy compared to baseline. Otherwise, both arms were equally effective in reducing duration, number, and intensity of attacks during three month of study. The results are shown in Figure 2.

## Discussion

Understanding migraine as a neuronal rather than vascular disease, have developed therapies with different mechanism of action. Cortical hyper-impulsiveness may play important role in the physiopathology of migraine thus, antiepileptic drugs can be used in prophylactic treatment of migraine attacks (7). Antiepileptic drugs are used in studies as a preventive treatment of migraine, recently. Yet, data on efficacy of pregabalin in this problem are lacking (22, 24). 

Pizzolato *et al.* in a 3-month follow-up study in 2011 propose that pregabalin may be effective and well tolerated in migraine prevention and may be an alternative preventive treatment of attacks. Limitations of their study were a small sample size (47 patients) and its design as an uncontrolled, open-label trial (24).

In 2010, Calandre *et al.* designed an open label study and publicized that Pregabalin use is related to significant decreases in headache frequency and severity, rescue medication intake, and HIT-6 scores (25).

Sadeghian and Motiei-Langroudi in 2014 compared the effectiveness of levetiracetam to placebo and valproate in 85 subjects. They followed subjects for 6 months and Efficacy was considered as a more than 50% decrease in the frequency of headaches. The results of their study have shown that levetiracetam has comparable efficacy to valproate in migraine prophylaxis (7).

Babazadeh *et al.* conducted a study to determine the effect of enalapril in preventing migraine headache attacks. They randomly assigned 70 patients aged 19-59 with 2-6 headache per months to two categories (enalapril 5 mg BID and valproate 200 mg BID). Each group received drugs for 8 weeks. Numbers and duration of attacks were assessed at the end of each month. The results of this study show that enalapril is more effective than valproate sodium in preventing migraine attacks (*P *< 0.001) (26).

In 2013, Zain *et al.* compared the efficacy and safety of topiramate and gabapentin in migraine prophylaxis in a 12-week randomized open label control trial. They desired outcome change in mean monthly migraine frequency and reduction in severity and average duration of an attack. Although both drugs were equally effective, gabapentin were better tolerated with patients (75% *vs.* 57.5%) (27).

In the present study, both sodium valproate and pregabalin reduced monthly headache duration, intensity and frequency with overall none statistically significant differences. Pregabalin and sodium valproate group were comparable in concern to change from baseline to month 3 in reducing average monthly migraine frequency, intensity, and duration. Based on our searches in database, no other studies are presented comparing pregabalin with sodium valproate, and this study is the first randomized double-blind placebo control trial to compare the effectiveness of pregabalin and sodium valproate on migraine attack prevention. 

## Conclusion

In conclusion, our study showed that pregabalin, has comparable efficacy with valproate sodium in reducing migraine frequency, intensity, and duration of attacks and could be an alternative for prophylaxis of migraine in whom suffering from side effects of valproate sodium.
